# Logical Reduction of Biological Networks to Their Most Determinative Components

**DOI:** 10.1007/s11538-016-0193-x

**Published:** 2016-07-14

**Authors:** Mihaela T. Matache, Valentin Matache

**Affiliations:** Department of Mathematics, University of Nebraska at Omaha, Omaha, NE 68182-0243 USA

**Keywords:** Boolean networks, Biological information theory, Mutual information, Sensitivity, Network reduction, Linear operators, Numerical simulations

## Abstract

Boolean networks have been widely used as models for gene regulatory networks, signal transduction networks, or neural networks, among many others. One of the main difficulties in analyzing the dynamics of a Boolean network and its sensitivity to perturbations or mutations is the fact that it grows exponentially with the number of nodes. Therefore, various approaches for simplifying the computations and reducing the network to a subset of relevant nodes have been proposed in the past few years. We consider a recently introduced method for reducing a Boolean network to its most determinative nodes that yield the highest information gain. The determinative power of a node is obtained by a summation of all mutual information quantities over all nodes having the chosen node as a common input, thus representing a measure of information gain obtained by the knowledge of the node under consideration. The determinative power of nodes has been considered in the literature under the assumption that the inputs are independent in which case one can use the Bahadur orthonormal basis. In this article, we relax that assumption and use a standard orthonormal basis instead. We use techniques of Hilbert space operators and harmonic analysis to generate formulas for the sensitivity to perturbations of nodes, quantified by the notions of influence, average sensitivity, and strength. Since we work on finite-dimensional spaces, our formulas and estimates can be and are formulated in plain matrix algebra terminology. We analyze the determinative power of nodes for a Boolean model of a signal transduction network of a generic *fibroblast* cell. We also show the similarities and differences induced by the alternative complete orthonormal basis used. Among the similarities, we mention the fact that the knowledge of the states of the most determinative nodes reduces the entropy or uncertainty of the overall network significantly. In a special case, we obtain a stronger result than in previous works, showing that a large information gain from a set of input nodes generates increased sensitivity to perturbations of those inputs.

**Electronic supplementary material:**

The online version of this article (doi:10.1007/s11538-016-0193-x) contains supplementary material, which is available to authorized users.

## Introduction

The past few decades have generated a large influx of data and information regarding a variety of real or artificial networks. The necessity to understand and use the data in a meaningful way has lead to various modeling approaches. In particular, Boolean network (BN) models introduced by Kauffman (1969) have acquired a significant importance in modeling networks where the node activity can be described by two states, 1 and 0, “ON and OFF,” “active and nonactive,” and where each node is updated based on logical relationships with other nodes. These models incorporate Boolean functions that are relevant to particular types of applications, such as signal transduction in cells (e.g., Helikar et al. 2008), genetic regulatory networks or other biological networks (e.g., Kauffman 1993; Shmulevich and Kauffman 2004; Shmulevich et al. 2002; Klemm and Bornholdt 2000; Albert and Othmer 2003), or neural networks (e.g., Huepe and Aldana 2002).

However, a simplification of the reality to binary states of the nodes does not ease the difficulty of studying large, complex networks for which the existing data may offer only partial information on the real interactions in the network and for which the dynamics are hard to study even under a deterministic approach. As a matter of fact, even smaller networks of only a few hundred nodes or less can pose serious difficulties in assessing the dynamics, given the exponential dependence of the state space on the number of nodes. Consequently, a number of approaches aiming at simplifying the computational difficulty of analyzing the dynamics have been proposed in recent years. For example, Goles et al. (2013) reduce the network and the number of updates needed to analyze the dynamics by generating sub-configurations of states that remain fixed regardless of the values of the other nodes and by identifying sets of updating schedules which have the same dynamics. They show that such networks are minimal in the sense that no edge can be deleted because every one of them represents a real interaction in the respective network. Various methods for reducing the network to a fairly small subset of nodes that are relevant for the long-term dynamics have been proposed. The definitions of “relevant” and “irrelevant” nodes differ depending on the actual approach. Some methods are related to eliminating stable nodes that end up in an attractor after a transient period and thus considered irrelevant. This may be paired with removing leaf nodes that do not contribute to the evolution of any other node, that is, with zero out-degree (outputs) like in Bilke and Sjunnesson (2001) or Richardson (2004), or with merging or collapsing mediator nodes with one in-degree (input) and one out-degree in Saadatpour et al. (2013). Other methods are based on eliminating irrelevant nodes that are frozen at the same value on every attractor together with nodes whose outputs go only to irrelevant nodes in Socolar and Kauffman (2003), Kaufman et al. (2005), or Kaufman and Drossel (2006). The basis of these methods is to reduce the “structure” of the network using some rules on the Boolean functions and then prove that such a reduction simplifies the identification of attractors. As expected, they may carry an intrinsic numerical burden. Furthermore, alternative methods for eliminating the need for complete enumeration of the states have been considered. For example,  Devloo et al. (2003) propose another formalism which permits calculation of the steady states as solutions of a system of steady-state equations, via an image function which identifies a state by its image under a certain function. The authors use constraint programming to solve the derived system of equations in an efficient way from a computational point of view.

In addition to the previously mentioned methods for network reduction, the entropy of the relevant components of the network which are comprised of relevant nodes that eventually influence each other’s state is used as a measure of uncertainty of the future behavior of a random state of the network by Krawitz and Shmulevich (2007a, (2007b). The entropy is a measure of uncertainty that has been used also by Ribeiro et al. (2008) to find the average mutual information of a random Boolean model of regulatory network as a way to quantify the efficiency of information propagation through the entire network. In this context, one needs to consider pairs of connected nodes and the intrinsic Boolean functions that govern the node updates, as opposed to evolving the networks in order to identify the attractors. Further research by some of the authors of Ribeiro et al. (2008), in particular Lloyd-Price et al. (2012), uses mutual information to test for a relationship between the robustness to perturbations of an attractor in a random BN and the amount of information propagated within the network when in that attractor. They found that there is a trade-off between robustness and information propagation and that at the edge of chaos, robustness is not correlated with information propagation.

On the other hand, the notions of entropy and mutual information have been long used as measures of complexity of dynamical systems, such as BNs, as described, for example, by Feldman and Crutchfield (1998) or by Sole and Luque (1997). Luque and Ferrera are concerned with the mutual information contained in random BNs and its behavior as the networks undergo their order–disorder phase transition, showing that the mutual information stored in the network has a maximum at the transition point.

Only recently the mutual information has been used as a method for identifying the most powerful and therefore relevant nodes in a BN, thus offering an efficient alternative approach to network reduction to a relevant subset of nodes Heckel et al. (2013). The mutual information, as a basic concept in information theory, allows one to represent the reduction in the uncertainty or entropy of the state of a node due to the knowledge of any of its inputs. A summation of all mutual information quantities over all nodes having a common input can be viewed as the determinative power of that input node. The more powerful the node, the more the information gain provided by the knowledge of its state. In Heckel et al. (2013), the authors use harmonic analysis to compare the determinative power of a set of inputs to the sensitivity to perturbations to those inputs showing that an input with large sensitivity need not have a large determinative power. On the other hand, large information gain from a set of inputs generates large sensitivity to perturbations of those inputs. Moreover, by considering the feedforward regulatory network of *E. coli*, it is shown that the knowledge of the states of the most determinative nodes reduces the uncertainty of the overall network significantly. Thus, one could focus on the dynamics of the reduced network of the nodes with the most determinative power.

In Heckel et al. (2013), the mutual information formula is obtained in terms of Fourier coefficients expressed in the Bahadur basis which assumes independence of the inputs of a Boolean function. In a subsequent paper by Klotz et al. (2014), it is shown that canalizing Boolean functions maximize the mutual information under the same assumption as in Heckel et al. (2013). This assumption is strong, since in a BN, there are correlations between inputs that build up as the network evolves in time. Our goal is to relax this assumption and allow dependence of inputs, while exploring the impact of a (necessarily) different basis on the results regarding the mutual information and the sensitivity to perturbations. We notice that some results still hold; however, not all are independent of the basis. At the same time we are interested to see the impact of our approach on the network reduction based on most determinative nodes of a specific biological network. In particular, we use a Boolean model of the signal transduction network of a generic *fibroblast* cell and we obtain results similar to Heckel et al. (2013).

In Sect. 2, we provide the basic definitions, the mathematical setup, and we use elements of operator theory to generate formulas for finding the sensitivity to perturbations of the nodes of the network, quantified by the concepts of influence, average sensitivity, and strength (to be defined in that section). We also discuss the computational aspects of using those formulas in applications. In Sect. 3, we provide formulas and estimates for mutual information, determinative power, and strength, paired with simulations, and estimates that link the mutual information and the sensitivity to perturbations. We also consider a special case that allows us to compare our analytical results to those in Heckel et al. (2013). Conclusions and further directions of research are in Sect. 4.

## Influence, Sensitivity, and Strength

In this section, we provide analytical formulas for the sensitivity to perturbations using a complete orthonormal basis that does not assume independence of the Boolean inputs. We pair this with some computational aspects regarding the application of the formulas to an actual biological network.

### Analytical Approach

Let $\Omega ^n = \{ 0, 1\} ^n$ and the random vector *X* valued in $\Omega ^n$. If *P* denotes the probability measure on the domain of definition of *X*, then denote $PX^{-1}$ the (cumulative) distribution of *X*. A BN is modeled as the set $[n]:=\{ 1,2, \ldots , n\}$ of *n* nodes, each node being ON (that is in state 1) or OFF (that is in state 0). Then any $\omega \in \Omega ^n$ is a possible state of the network. Each node $i \in [n]$ has an associated Boolean function $f_i:\Omega ^n\rightarrow \Omega$ that governs the dynamics of the node. We are usually interested in how the network evolves by iterating the map $F = (f_1, f_2, \ldots , f_n)$ a large number of times. Although the measures used in this paper are discrete, we use notation typical for measure theoretical arguments to write shorter and more elegant proofs.

Given a node *i* with Boolean function $f_i:\Omega ^n\rightarrow \Omega$, the influence of the *j*th input on $f_i$ has been formulated in various ways in the literature. Following the authors of Shmulevich and Kauffman (2004), Kahn et al. (1988), and Ghanbarnejad and Klemm (2012), we recall the following:

#### Definition 1

The *influence*, $I_j(f_i)$, of variable $x_j$ on the Boolean function $f_i$, or equivalently, the activity of node *j* on node *i*, is defined as follows:

1 $$\begin{aligned} I_j(f_i):=P(f_i(X)\ne f_i(X\oplus e_j)) \end{aligned}$$

where $X\oplus e_j$ is the random vector obtained by flipping the *j*th slot of *X* from 1 to 0 or viceversa. The *average sensitivity*, $\mathrm{avs}(f_i)$, of $f_i$ is the sum of its incoming activities

2 $$\begin{aligned} \mathrm{avs}(f_i):=\sum _{j=1}^nI_j(f_i). \end{aligned}$$

The *strength*, $\sigma (f_i)$, of $f_i$ is the sum of the outgoing activities

3 $$\begin{aligned} \sigma (f_i):=\sum _{j=1}^nI_i(f_j). \end{aligned}$$

Alternatively, $I_j(f_i)$ can be regarded as the average of the Boolean partial derivative $\partial ^{(j)}f_i(X) = \delta _{f_i(X), f_i(X\oplus e_j)}$ with respect to the probability measure *P* as specified in Ghanbarnejad and Klemm (2012). Here $\delta$ is Kronecker’s delta function which is equal to one if the two variables are equal and zero otherwise. The definition is originally introduced in the context of assuming the state of the BN as a random vector $X=(X_1, \ldots ,X_n)$ with independently distributed coordinates, but this property plays no role in it. However, the aforementioned property plays an essential role in papers like Heckel et al. (2013), Klotz et al. (2014), or Kahn et al. (1988) where it is essential in obtaining formulas for $I_j(f_i)$ (and other related quantities relevant in the study of BNs) in terms of the Fourier coefficients of $f_i$ [see, e.g., (Kahn et al. 1988, Lemma 4.1)]. The Hilbert space where those formulas are obtained is $L^2(\Omega ^n, dPX^{-1})$. The complete orthonormal basis used in Kahn et al. (1988) is the, so-called, Bahadur basis (see Heckel et al. 2013). In order for that family of functions to form a complete orthonormal basis of $L^2(\Omega ^n, dPX^{-1})$ it is necessary that $X_1, \ldots , X_n$ be independently distributed. However, the nodes of a BN may mutually influence each other, so independence is a restrictive assumption.

A composition operator is an operator on a linear space $\mathcal {S}$ of functions defined on a set *E*. For any fixed self-map $\varphi$ of *E*, the operator

$$\begin{aligned} C_\varphi f:=f\circ \varphi \qquad f\in \mathcal {S} \end{aligned}$$

is called the composition operator with symbol $\varphi$ or induced by $\varphi$. It is necessarily linear. We will use such operators on $\mathcal {S}=L^2(\Omega ^n, dPX^{-1})$.

Let $\varphi _j$ be the *j*th slot flip map. This means that, for all $\omega \in \Omega ^n, \varphi _j(\omega )$ is the Boolean vector in $\Omega ^n$ obtained by flipping the *j*th coordinate of $\omega$. Observe that $\varphi _j$ are obviously self-inverse and hence so are the composition operators they induce, a fact which will be used without further comments throughout this paper. In the following, $\langle \, ,\, \rangle$ denotes the usual inner product of $L^2(\Omega ^n, dPX^{-1})$ and $\Vert \quad \Vert$ the norm induced by that inner product. Also, $T^*$ is the notation used for the adjoint of any operator *T*, whereas *I* denotes the identity operator. With these notations we prove:

#### Proposition 1

For all Boolean functions $f_i$, the following formulas hold:

4 $$\begin{aligned} I_j(f_i)= & {} \langle (I-C_{\varphi _j})^*(I-C_{\varphi _j})f_i, f_i\rangle \qquad j=1, \ldots ,n\end{aligned}$$

5 $$\begin{aligned} \mathrm{avs}(f_i)= & {} \langle Tf_i, f_i\rangle \qquad \mathrm{where}\qquad T=\sum _{j=1}^n(I-C_{\varphi _j})^*(I-C_{\varphi _j})\end{aligned}$$

6 $$\begin{aligned} \sigma (f_i)= & {} \sum _{j=1}^n\langle T_if_j, f_j\rangle \qquad \mathrm{where}\qquad T_i=(I-C_{\varphi _i})^*(I-C_{\varphi _i}) \end{aligned}$$

#### Proof

Using a well-known change in measure formula, for any Boolean function *f* (we drop the index for simplicity of notation) one can write

$$\begin{aligned} I_j(f)= & {} P(\{ f(X)\ne f(X\oplus e_j)\})=\int |f(X)- f(X\oplus e_j)|^2\, dP\\= & {} \int |f(X)- f\circ \varphi _j(X)|^2\, dP=\int _{\Omega ^n}|f- f\circ \varphi _j|^2\, dPX^{-1}\\= & {} \Vert (I-C_{\varphi _j})f\Vert ^2=\langle (I-C_{\varphi _j})^*(I-C_{\varphi _j})f, f\rangle . \end{aligned}$$

$$\square $$

#### Proposition 2

For all $j=1,2, \ldots ,n$, let $\Lambda _j$ denote the largest eigenvalue of $T_j=(I-C_{\varphi _j})^*(I-C_{\varphi _j})$, respectively, whereas $\Lambda$ is the largest eigenvalue of *T*. The following estimates hold:

7 $$\begin{aligned} I_j(f)\le \Lambda _j\, E[f(X)]\end{aligned}$$

8 $$\begin{aligned} \mathrm{avs}(f)\le \Lambda \, E[f(X)]. \end{aligned}$$

Hence:

9 $$\begin{aligned} \sigma (f_i)\le \Lambda _iE[F(X)], \quad i\in [n], \end{aligned}$$

where $$F=\sum _{j=1}^nf_j$$.

#### Proof

Indeed, both the operators $T_j$ and *T* are nonnegative operators, and hence, their numerical range is equal to the line interval with endpoints the least, respectively, the largest eigenvalue. On the other hand, formulas (4) and (5) show that $I_j(f)/\Vert f\Vert ^2$ and $\mathrm{avs}(f)/\Vert f\Vert ^2$ belong to the numerical range of the operator $T_j$, respectively, *T*. Combining all that with the fact that *f*, being a Boolean function, satisfies condition $E[f(X)]=\Vert f\Vert ^2$, proves (7) and (8).

For arbitrary fixed $i\in [n]$ one has by (7) that

$$\begin{aligned} \sigma (f_i)\le \Lambda _i\sum _{j=1}^nE[f_j(X)]. \end{aligned}$$

Given that obviously $\sum _{j=1}^nE[f_j(X)]=E[F(X)]$, estimate (9) follows.

$$\square $$

The space $L^2(\Omega ^n, dPX^{-1})$ has a simple and natural complete orthonormal basis, namely $\mathcal {B}=\{ e_\omega =\chi _{\omega }/ \sqrt{p_{\omega }}:\omega \in \Omega ^n\}$, where

$$\begin{aligned} \chi _{\omega }(x)=\delta _{\omega ,x}\qquad x\in \Omega ^n \end{aligned}$$

and

$$\begin{aligned} p_{\omega }=P(X=\omega ) \qquad \omega \in \Omega ^n. \end{aligned}$$

We assume all states are possible, so $p_{\omega } > 0$.

Checking the fact that $\mathcal {B}$ is a complete orthonormal basis of $L^2(\Omega ^n, dPX^{-1})$, whether $X_1,\ldots ,X_n$ are independently distributed or not is left to the reader.

#### Proposition 3

For all $j=1, \ldots , n$, the operator $T_j=(I-C_{\varphi _j})^*(I-C_{\varphi _j})$ has a matrix with respect to $\mathcal {B}$ whose entries are

10 $$\begin{aligned} a_{\omega ,\eta }(T_j)= & {} \delta _{\omega ,\eta }-\sqrt{\frac{p_{\varphi _j(\omega )}}{ p_\omega }}\delta _{\varphi _j(\omega ),\eta } -\sqrt{\frac{p_{\varphi _j(\eta )}}{ p_\eta }}\delta _{\varphi _j(\eta ),\omega }\nonumber \\&\quad +\sqrt{\frac{p_{\varphi _j(\omega )}p_{\varphi _j(\eta )}}{ p_\omega p_\eta } } \delta _{\varphi _j(\omega ),\varphi _j(\eta ) }\qquad \omega ,\eta \in \Omega ^n. \end{aligned}$$

Hence, the entries in the matrix of the operator *T* are:

11 $$\begin{aligned} a_{\omega , \eta }(T)= \sum _{j=1}^n a_{\omega ,\eta }(T_j) \qquad \omega \in \Omega ^n. \end{aligned}$$

#### Proof

Given $\omega ,\eta \in \Omega ^n$, the entry $a_{\omega ,\eta }(T_j)$ in the matrix of $(I-C_{\varphi _j})^*(I-C_{\varphi _j})$ is

12 $$\begin{aligned} a_{\omega ,\eta }(T_j)=\langle (I-C_{\varphi _j})^*(I-C_{\varphi _j})e_\eta ,e_\omega \rangle =\langle (I-C_{\varphi _j})e_\eta , (I-C_{\varphi _j})e_\omega \rangle . \end{aligned}$$

Note that

$$\begin{aligned} C_{\varphi _j}\chi _\nu =\chi _{\varphi _j(\nu )} \qquad \nu \in \Omega ^n, \end{aligned}$$

and hence

13 $$\begin{aligned} C_{\varphi _j}e _\nu =\sqrt{\frac{p_{\varphi _j(\nu )}}{p_\nu }}e_\nu \qquad \nu \in \Omega ^n. \end{aligned}$$

Equalities (12) and (13) combine into establishing by a straightforward computation Eq. (10).

$$\square $$

Therefore, one can state the following:

#### Corollary 1

Given a Boolean function *f*, the following practical formulas hold:

14 $$\begin{aligned} I_j(f)=\sum _{\omega ,\eta \in \Omega ^n}a_{\omega ,\eta }(T_j)f(\omega )f(\eta )\sqrt{p_\omega p _\eta } \end{aligned}$$

and

15 $$\begin{aligned} \mathrm{avs}(f)=\sum _{\omega ,\eta \in \Omega ^n}a_{\omega ,\eta }(T)f(\omega )f(\eta )\sqrt{p_\omega p_\eta }. \end{aligned}$$

Hence:

16 $$\begin{aligned} \sigma (f_i)=\sum _{j=1}^n\sum _{\omega ,\eta \in \Omega ^n}a_{\omega ,\eta }(T_i)f_j(\omega )f_j(\eta )\sqrt{p_\omega p _\eta }, \quad i\in [n]. \end{aligned}$$

Indeed, (14) and (15) are immediate consequences of the matricial description of operators $T_j$ and *T* combined with the following computation of the Fourier coefficients $c_\omega , \omega \in \Omega ^n$ of *f* relative to $\mathcal {B}$:

17 $$\begin{aligned} c_\omega =\langle f, e_\omega \rangle =\frac{1}{\sqrt{p_\omega }}\int _{\Omega ^n}f\chi _\omega dPX^{-1}=\frac{1}{\sqrt{p_\omega }}f(\omega )P(X=\omega )=f(\omega )\sqrt{p_\omega }. \end{aligned}$$

#### Example 1

In this example, we show that formula (14) agrees with the definition of $I_j(f)$. To this goal, assume a product distribution, which is the basic assumption of Heckel et al. (2013), such that every state of the network is equally likely. Thus, the probability of any state is $1/2^n$. Then

$$\begin{aligned} I_j(f)= & {} \sum _{\omega ,\eta \in \Omega ^n}a_{\omega ,\eta }(T_j)f(\omega )f(\eta )\sqrt{p_\omega p _\eta } = \frac{1}{2^n}\sum _{\omega ,\eta \in \Omega ^n}a_{\omega ,\eta }(T_j)f(\omega )f(\eta )\nonumber \\ \quad= & {} \frac{1}{2^n}\sum _{\omega ,\eta \in {\mathrm{supp}}\, f} \left[ \delta _{\omega ,\eta }-\delta _{\varphi _j(\omega ),\eta } - \delta _{\varphi _j(\eta ),\omega }+\delta _{\varphi _j(\omega ),\varphi _j(\eta ) }\right] \end{aligned}$$

where ${\mathrm{supp}}\, f$ is the support $f^{-1}(1)$ of the function *f*.

Let *f* be the Boolean function with support ${\mathrm{supp}}\, f = \{(0,1,1), (1,1,1)\}$. Then obviously a flip of $x_1$ does not generate a flip in the output, so $I_1(f) = 0$. Similarly, a flip in $x_2$ generates a flip of the output only for ($x_1, x_2, x_3$) = (0,1,1), (0,0,1), (1,0,1) or (1,1,1), so, by definition, $I_2(f) = 4/2^3 = 1/2$. By symmetry, $I_3(f) = 1/2$. On the other hand,

$$\begin{aligned}&I_1(f)= \frac{1}{8}[a_{(0,1,1),(0,1,1)}(T_1) + a_{(0,1,1),(1,1,1)}(T_1) + a_{(1,1,1),(0,1,1)}(T_1) \nonumber \\&\qquad \qquad \quad +\, a_{(1,1,1),(1,1,1)}(T_1)] = \frac{1}{8}[2 + (-2) + (-2) + 2] = 0 \end{aligned}$$

and similarly

$$\begin{aligned} I_2(f)= \frac{1}{8}[ 2 + 0 + 0 + 2] = \frac{1}{2} \end{aligned}$$

so one can see that formula (14) agrees with the definition of $I_j(f)$.

In order to compute the influence of each node in the network on all its output nodes (those to which the node under consideration is an input), we generate MATLAB codes that involve nested “for loops.” Note that, if we denote by $k_i$ the actual number of inputs to node *i* (its connectivity), for formula (14) there are $i \times k_i \times 2^{2k_i}$ such loops. This exponential number of loops can easily make the computations prohibitive. As a matter of fact, in the actual simulations, even a single connectivity of at least 12 nodes turned out to be excessive for the capabilities of MATLAB. Thus, one has to rely either on easier estimates, such as the upper bounds of Proposition 2, or find alternative exact formulas. However, a quick analysis indicates that to compute the upper bounds one would still need exponentially many “for loops,” since the procedure would require again the construction of the matrices $T_j$ and *T*. For this reason, in the sequel we use the following equivalent formula which follows from the definition.

Recall that, for any Boolean function $f, {\mathrm{supp}}\, f$ denotes the support of *f*, that is, ${\mathrm{supp}}\, f =f^{-1} (1)$.

#### Remark 1

The following formula holds

18 $$\begin{aligned} I_j(f)= \sum _{\omega \in {\mathrm{supp}}\, f\setminus \varphi _j({\mathrm{supp}}\, f)}\left( {p_\omega }+{p_{\varphi _j(\omega )}}\right) , \end{aligned}$$

where the sum above is considered 0 if ${\mathrm{supp}}\, f\setminus \varphi _j({\mathrm{supp}}\, f)=\emptyset$.

Hence:

#### Corollary 2

Let $f_i, i\in [n]$ be the Boolean update function of node *i*. Then

19 $$\begin{aligned} \mathrm{avs}(f_i)=\sum _{j=1}^n\left( \sum _{\omega \in {\mathrm{supp}}\, f_i\setminus \varphi _j({\mathrm{supp}}\, f_i)}\left( {p_\omega }+{p_{\varphi _j(\omega )}}\right) \right) \quad i\in [n]\end{aligned}$$

20 $$\begin{aligned} \sigma (f_i)=\sum _{j=1}^n\left( \sum _{\omega \in {\mathrm{supp}}\, f_j\setminus \varphi _i({\mathrm{supp}}\, f_j)}\left( {p_\omega }+{p_{\varphi _i(\omega )}}\right) \right) \quad i\in [n]. \end{aligned}$$

Observe that:

#### Remark 2

Formula (18) is computationally efficient because it identifies the influential nodes, namely those in the support of the given Boolean function which get mapped outside of support by that function. In particular, the variable $x_j$ has null influence on the Boolean function *f*, that is $I_j(f)=0$, if and only if $\varphi _j({\mathrm{supp}}\, f)\subseteq {\mathrm{supp}}\, f$.

Indeed, $$\varphi _j({\mathrm{supp}}\, f)\subseteq {\mathrm{supp}}\, f$$ implies

$$\begin{aligned} {\mathrm{supp}}\, f=\varphi _j(\varphi _j({\mathrm{supp}}\, f))\subseteq \varphi _j({\mathrm{supp}}\, f)\subseteq {\mathrm{supp}}\, f. \end{aligned}$$

This agrees perfectly with the definition (1) of the influence as the probability of a change in the output of *f* when its *j*th input is flipped.

To understand the computational efficiency of formula (18), or equivalently the definition of the influence, we run MATLAB codes for both formula (14) and (18) on the same network, and compare the processing times. The results are included in Supplementary Material, Section 1. We present one more example.

#### Example 2

We call a BN: “Dominant states network (DSN)” if the update Boolean functions of nodes are characteristic functions of distinct states (called dominant states), that is if there is a set of *n* states $S=\{ \omega _1,\ldots , \omega _n\}$, so that $f_1=\chi _{\omega _1}, \ldots , f_n=\chi _{\omega _n}$.

Our previous considerations and formulas show by straightforward computations that, in the case of a DSN, one has that:

$$\begin{aligned} \sigma (f_i)=\sum _{j=1}^n\left( {p_{\omega _j}}+{p_{\varphi _i(\omega _j)}}\right) \quad i\in [n] \end{aligned}$$

and

$$\begin{aligned} \mathrm{avs} (f_i)=\sum _{j=1}^n\left( {p_{\omega _i}}+{p_{\varphi _j(\omega _i)}}\right) \quad i\in [n]. \end{aligned}$$

## Determinative Power and Strength

In this section, we are comparing the impact of node strength to the so-called determinative power of nodes defined and explored in Heckel et al. (2013) under the assumption of a BN with product distribution of states. We recall the main definitions and concepts from Heckel et al. (2013) and Cover and Thomas (2006). These include the notion of entropy of random variables, which is a measure of uncertainty, and the mutual information which is a measure of dependence between two random variables and is defined in terms of the entropy.

### Definition 2

Let *X* and *Y* be discrete random variables. The *entropy* of *X* is defined as

$$\begin{aligned} H(X) = -\sum _x p_x\log _2p_x = -E[\log _2(X)] \end{aligned}$$

which in binary reduces to the function

$$\begin{aligned} h(p) = -p\log _2(p) - (1-p)\log _2(1-p), \qquad p = P(X = 1). \end{aligned}$$

The *conditional entropy* of *Y* conditional on the knowledge of *X* is

$$\begin{aligned} H(Y|X) = \sum _xp_xH(Y|X=x) = -E[\log _2P(Y|X)]. \end{aligned}$$

The *mutual information* (MI) is the reduction in uncertainty of the random variable *Y* due to the knowledge of *X*. That is

$$\begin{aligned} \mathrm{MI}(Y;X) = H(Y) - H(Y|X). \end{aligned}$$

In principle, the mutual information is a measure of the “gain of information,” or the *determinative power* (DP) of *X* over *Y*. The authors of Heckel et al. (2013) use the MI to construct the DP of a node *j* over the states of a BN, namely

21 $$\begin{aligned} DP(j) = \sum _{i=1}^nMI(f_i(X);X_j) \end{aligned}$$

which represents a summation of all “information gains” obtained from node *j* over its outputs (i.e., nodes that have *j* as an input). Besides providing a number of related mathematical results to which we will refer below, the authors identify the nodes with the largest determinative power in a feedforward *E. coli* network, assuming a product distribution of the input states. The goal is to be able to reduce the network to a smaller sub-network whose knowledge can provide sufficient information about the entire network; in other words the entropy of the network conditional on the knowledge of this sub-network is small enough. The authors show that by considering the nodes with most DP one can reduce the network to less than a half of its original size, so that for larger sub-networks the entropy does not improve significantly once an approximate (threshold) network size is reached.

As specified in Introduction, network reduction is an important topic in the literature, since many real networks have sizes that lead to prohibitive computations and manipulations as we can see in Supplementary Material with the computation of the influence. For instance, signal transduction networks such as that of a generic *fibroblast* cell, which we will use as an example, can have thousands of nodes that are interconnected. The analysis and dynamical study of such networks becomes prohibitive due to the computational burden despite the advances in technology and data science. Thus, finding meaningful ways to reduce the network to a significant “core” or “relevant component” has been of interest for a number of authors, and a number of procedures have been proposed. Ultimately, all of them generate a clear trade-off between accuracy and computational burden.

We are interested in comparing the effect of network reduction applied to the *fibroblast* network, by considering the nodes with largest DP on the one hand, and the nodes with largest strength values on the other hand. Before we compare them, let us focus on some theoretical results that supplement some of the formulas in Heckel et al. (2013) for the less restrictive case we consider in this paper, that is, not requiring a product distribution of the input values.

If *X* is the state of the network with values in $\Omega ^n$, let $X_A$ taking values in $\Omega ^{|A|}$ be the collection of states of the nodes in set $A \subseteq [n]$. So *X* can be written as $(X_A, X_{A^c })$, where $A^c = [n]\setminus A$. Let $p_{\omega |\omega _A} = P(X = \omega | X_A = \omega _A)$.

### Theorem 1

The following formula for conditional entropy holds

22 $$\begin{aligned} H(f(X)|X_A) = E_A\left[ h\left( \sum _{\omega \in {\mathrm{supp}}\, f}\, p_{\omega | X _A}\right) \right] \end{aligned}$$

where $E_A$ refers to expected value with respect to the marginal distribution of $X_A$.

### Proof

By the definition of the conditional entropy

$$\begin{aligned} H(f(X)|X_A)=\sum _{\omega _A \in \Omega ^{|A|}}P(X_A=\omega _A)H(f(X)|X_A=\omega _A) \end{aligned}$$

which in our binary case reduces to

$$\begin{aligned} H(f(X)|X_A)=\sum _{\omega _A \in \Omega ^{|A|}}P(X_A=\omega _A)h(P(f(X) = 1|X_A=\omega _A)) \end{aligned}$$

and the obvious equality $P(f(X)=1|X_A=\omega _A)=E[f(X)|X_A=\omega _A]$ implies

23 $$\begin{aligned} H(f(X)|X_A)= & {} \sum _{\omega _A \in \Omega ^{|A|}}P(X_A=\omega _A)h(E[f(X)|X_A=\omega _A])\nonumber \\= & {} E_A[h(E[f(X)|X_A])]. \end{aligned}$$

But

$$\begin{aligned} E[f(X)|X_A=\omega _A] = \sum _{\omega \in \Omega ^n}\, f(\omega )P(X = \omega | X_A = \omega _A) = \sum _{\omega \in {\mathrm{supp}}\, f}\, p_{\omega | \omega _A} \end{aligned}$$

which is a number in [0, 1] and we can substitute it in (23) to get formula (22).

$$\square $$

Formula (22) is exactly the analog of Theorem 1 of Heckel et al. (2013) where it is written for a system with states $-1$ and 1 as opposed to 0 and 1.

### Proposition 4

The mutual information formula $MI(f(X);X_A)$ can be written as

24 $$\begin{aligned} MI(f(X); X_A) = h\left( \sum _{\omega \in {\mathrm{supp}}\, f}\, p_{\omega }\right) - E_A\left[ h\left( \sum _{\omega \in {\mathrm{supp}}\, f}\, p_{\omega | X _A}\right) \right] . \end{aligned}$$

### Proof

The formula for MI follows again directly from the definition of the mutual information

$$\begin{aligned} MI(f(X); X_A) = H(f(X)) - H(f(X)|X_A) = h(E[f(X)]) - H(f(X)|X_A). \end{aligned}$$

But

$$\begin{aligned} h(E[f(X)]) = h\left( \sum _{\omega \in \Omega ^n}\, f(\omega ) P(X = \omega )\right) = h\left( \sum _{\omega \in {\mathrm{supp}}\, f}\, p_{\omega }\right) \end{aligned}$$

with the argument of *h* being in [0, 1] as needed. Substituting this and formula (22) in the definition of the mutual information we obtain formula (24).

$$\square $$

Now we focus on two special (extreme) cases considered in Heckel et al. (2013), to see if we can identify an analog of the results in that paper, where the authors use the additional assumption of independence paired with the Bahadur basis (a family of functions which form a complete orthonormal basis of $L^2(\Omega ^n, dPX^{-1})$ provided that $X_1, X_2, \ldots , X_n$ are independent).

**Special cases:**

- $$A = [n]$$. Then $$A^c = \emptyset $$ and
  $$\begin{aligned} MI(f(X); X) = h\left( \sum _{\omega \in {\mathrm{supp}}\, f}\, p_{\omega }\right) \end{aligned}$$
  which is maximized for $\sum _{\omega \in {\mathrm{supp}}\, f}\, p_{\omega } = 1/2$, in other words if $E[f(X)] = P(f(X) = 1) = 1/2$. Hence, we are dealing with a nonbiased function. So the closer the function *f* is to a nonbiased function, the larger the MI between its output and all of its inputs. This is similar to what was observed in Heckel et al. (2013).
- $A = \{ i\}$ where *i* is a fixed input/node. Thus, $\omega _A = \omega _i, X_A = X_i$. The mutual information can be written as
  $$\begin{aligned} MI(f(X); X_i)= & {} h\left( \sum _{\omega \in {\mathrm{supp}}\, f}\, p_{\omega }\right) - P(X_i = 1) h\left( \sum _{\omega \in {\mathrm{supp}}\, f}\, p_{\omega | 1}\right) \nonumber \\&\quad - P(X_i = 0) h\left( \sum _{\omega \in {\mathrm{supp}}\, f}\, p_{\omega | 0}\right) \end{aligned}$$
  In comparison with Heckel et al. (2013), this formula does not allow for a simplification to a small subset of Fourier coefficients in order to find the MI. In their formula, the authors of Heckel et al. (2013) identify three coefficients that act as independent variables. Based on them they manage to obtain some information on the behavior of the MI, which is not the case for our approach. The reason for considering this special case is that the quantity $\mathrm{MI}(f(X); X_i)$ is also known as information gain, or informativeness, and is common in information theory. Thus, we note that a change in the underlying basis can induce different results and situations.

Now that we have a deeper understanding of the MI as used in formula (21), let us turn to the network under consideration, namely the signal transduction network of a generic *fibroblast* cell which consists of several main signaling pathways, including the receptor tyrosine kinase, the G-protein coupled receptor, and the integrin signaling pathway. A Boolean representation of this network has been provided in Helikar et al. (2008), and has been studied further in Kochi and Matache (2012) and Kochi et al. (2014). The fully annotated signal transduction model is freely available for simulations and/or download via the Cell Collective software from www.thecellcollective.org Helikar et al. (2012) and Helikar et al. (2013). Each node in the model represents a signaling molecule (mainly protein). The network has 130 nodes with connectivities that vary between 1 and 14 nodes.

**Fig. 1:**
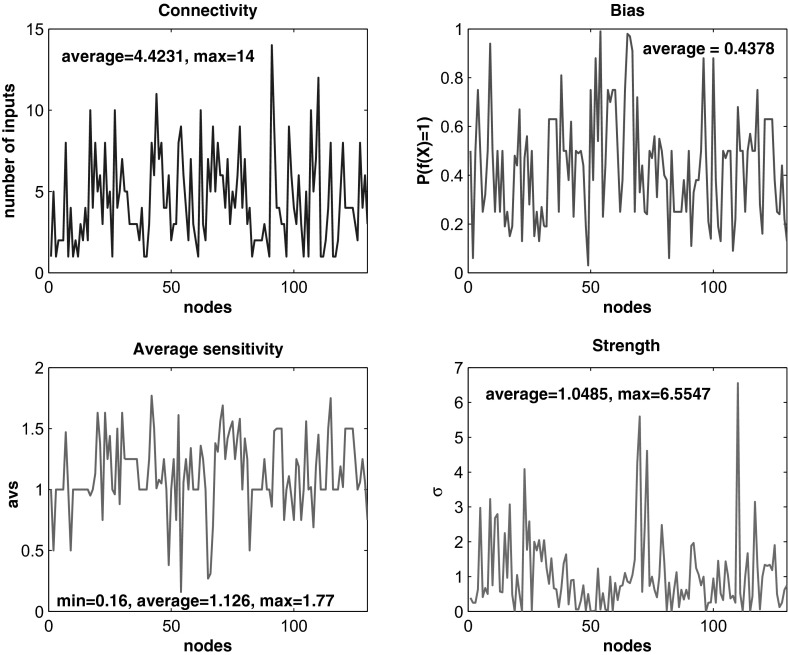
(Color Figure online) Main numerical characteristics of the nodes of the *fibroblast* network as specified in each subplot

Using formula (18), one can quickly determine the average sensitivity and the strength of all nodes in the *fibroblast* network. In Fig. 1, we plot them against the nodes (in alphabetic order), together with two more plots on the connectivity and the bias, i.e., $P(f(X) = 1)$, for an overall view of the main numerical characteristics of the *fibroblast* nodes.

**Fig. 2:**
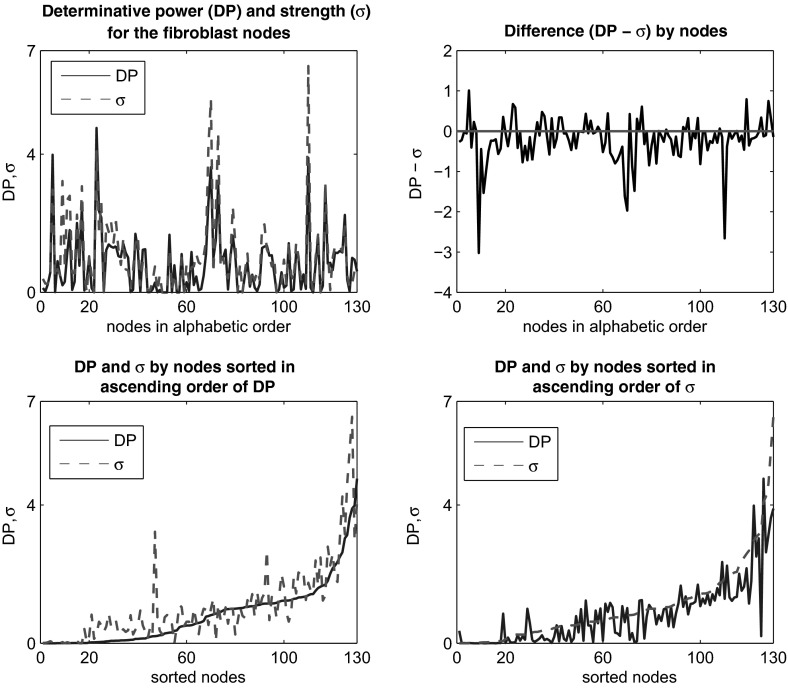
(Color Figure online) Comparison of DP and $\sigma$ by nodes of the *fibroblast* network. The nodes are sorted by names in the *top panels*, while in the *bottom panels* they are ordered according to increasing DP, and $\sigma$, respectively, as indicated in the graphs. Note that the strength values seem to be slightly larger than the DP values

We compute the DP of the nodes in the *fibroblast* network and compare them with the node strength $\sigma$. The results are shown in Fig. 2. For the network under consideration, the strength values seem to be slightly larger than the DP values. We have conducted a statistical analysis related to DP and $\sigma$ values for the *fibroblast* network. In summary, there is enough statistical evidence that the average DP-$\sigma$ is negative with a *p* value of basically zero. The paired test gives an upper bound of $-0.14208$ for a 95 % confidence interval for the difference DP$-\sigma$. On the other hand, a linear regression analysis indicates a fairly strong linear relationship between the two variables with a 75.1 % coefficient of determination (COD), and a higher COD of 82.4 % for the linear relationship between the average $\sigma$ and the number of outlinks corresponding to the nodes. The average values are computed over all nodes with a given number of outlinks. This relationship is weaker for average DP versus number of outlinks with a COD of 60.3 %. We also note that the outliers occur mostly for nodes with a larger number of outlinks. In other words, fewer outlinks generate a stronger correlation between the DP or $\sigma$ and the number of outlinks. For example, there is one particular node in the network, namely *EGFR*, that generates the maximum DP and is the only node with 13 outlinks. If we eliminate this node from the correlation analysis, the COD for average DP versus outlinks increases from 60.3 to 81.3 %. Notably, mutations of the *EGFR*, epidermal growth factor receptor, are known to be related to lung cancer, interfering with the signaling pathways within the cell triggered to promote cell growth and division (proliferation) and cell survival. The second node in the order of DP is *ASK1*, apoptosis signal-regulating kinase 1, and plays important roles in many stress-related diseases, including cancer, diabetes, cardiovascular, and neurodegenerative diseases. The third node is *Src*, proto-oncogene tyrosine-protein kinase, which is involved in the control of many functions, including cell adhesion, growth, movement, and differentiation. The fourth node is *PIP3_345*, phosphatidylinositol (3,4,5)-trisphosphate that functions to activate downstream signaling components, while the fifth node is *PKC*, protein kinase C, involved in receptor desensitization, in modulating membrane structure events, in regulating transcription, in mediating immune responses, in regulating cell growth, and in learning and memory. The DP procedure managed to capture the importance of these nodes in relationship to the rest of the network. Four of the top five DP nodes are also among the five strongest nodes which are: *Src*, *PIP3_345*, *PKC*, *PIP2_45*, and *EGFR*. Thus, the strength also captures biologically important nodes. Moreover, higher DP and strength values are correlated with a larger number of outlinks as seen from the figures, which means that this procedure can identify hubs in the network. It is also apparent from the figures that the COD increases when considering smaller DP and $\sigma$ values. We have included relevant figures in Supplementary Material, Sect. 2

We would also like to point out at this time that the MI has been used as way to identify relevant pairs of genes in genetic expression data sets by Butte and Kohane (2000, (2003), and Jiang et al. (2009). Those authors identify relevance networks by selecting pairs of genes whose MI surpasses a given threshold. For example, in Butte and Kohane (2000) it is shown that the relevance networks are of the following types: those that link identical genes, those linking genes with similar functions, those linking genes in the same biological pathway, and combinations of these. In our work, the MI is directed from the node toward its outputs via the outlinks, so we do not involve the bidirectional aspect of the previous studies mentioned here. Moreover, we do not use genetic expression data sets, but actual Boolean functions. Nevertheless, it will be of interest to explore in the future the main types or classes of nodes/sub-networks identified by the DP procedure in a variety of cellular networks.

Next, let us compute the network entropy generated by sub-networks chosen based on top DP or strength values of nodes. If we denote by $A_l$ the collection of the top *l* nodes in order of DP or $\sigma$, then we compute

25 $$\begin{aligned} H(X|X_{A_l}) \le \sum _{i=1}^nH(X_i|X_{A_l}), \qquad \hbox {for} \quad l = 1, 2, 3, \ldots , n. \end{aligned}$$

**Fig. 3:**
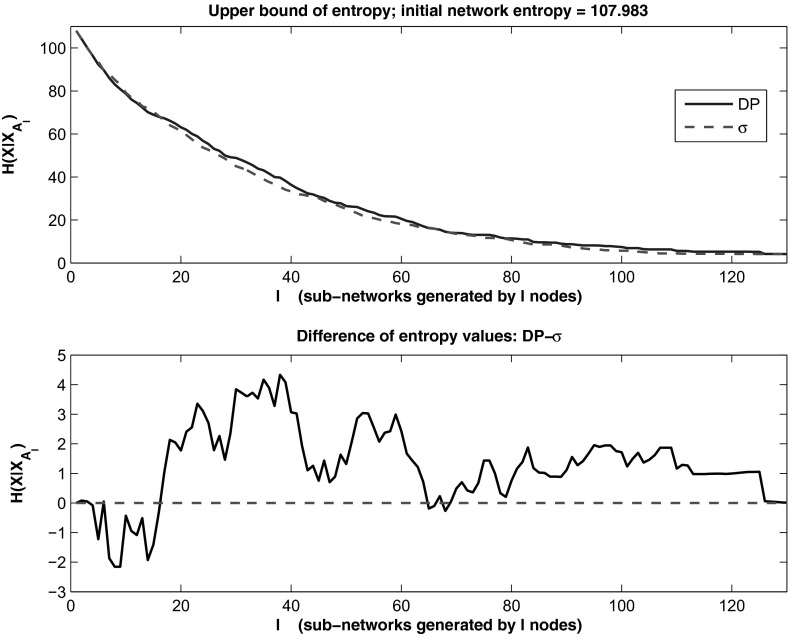
(Color Figure online) Values of the *upper* bound in (25) for sub-networks chosen based on the *top* *l* values of DP and $\sigma$, respectively. The *bottom panel* shows the differences in the entropy that favor mostly $\sigma$ for $l > 20$ with approximation

We plot the values of the larger quantity in (25) against *l* and obtain the graph in Fig. 3, where we note that the entropy decreases with increased sub-network size *l*, and that the two curves are very close, with a slightly better result for $\sigma$ which provides lower entropy values for most values of *l*, as seen from the bottom panel where we plot also the differences of entropy values by *l*. As the sub-network size increases, the strength provides a somewhat tighter upper bound for the entropy of the network. Note that the actual entropy will be smaller than the upper bound graphed in Fig. 3, and that sub-networks of sizes 60 or more (with approximation), do not yield a significant improvement of the entropy. Thus, it suffices to consider less than half of the original network to be able to predict the overall network behavior with fairly low uncertainty/entropy levels.

Knowing that the DP method allows a network reduction without a significant increase in entropy, one could use a tested approach for node elimination that preserves essential dynamical properties to reduce the network to the sub-network of the top DP nodes. For example, in Naldi et al. (2009b) the authors introduce a general method for eliminating nodes one by one by basically connecting directly the inputs of a removed node to its output nodes. Of course, one needs to either keep or consider some extra decisions on autoregulated nodes, that is nodes that are or may become self-inputs upon elimination of other nodes. One also needs to understand the impact of the order in which nodes are removed. In our case, the natural order is provided by the sorted DP values. In Naldi et al. (2009b), it is shown that with their approach the attractors and stable states are preserved. We note here that there are nodes whose removal may have no impact on the dynamics, like those with no outputs. Moreover, the authors of Naldi et al. (2009b) have developed a Java software Naldi et al. (2009a) that allows one to apply the reduction algorithm and analyze attractors. Alternative methods have been proposed by Veliz-Cuba and collaborators in Veliz-Cuba (2011); Veliz-Cuba et al. (2015) that could be used in conjunction with the DP method. Future work will explore those methods.

One could actually go one step further and provide the following estimates for the conditional entropy $H(f(X)| X_A)$, which are the analog of Theorem 2 of Heckel et al. (2013).

### Theorem 2

The following estimates of the conditional entropy hold:

26 $$\begin{aligned} LB \le H(f(X) | X_A) \le LB ^{1/{\ln 4}} \end{aligned}$$

where

27 $$\begin{aligned} \mathrm{LB}= & {} 4\left( E[f(X)] - E_A[E[f(X) | X_A]^2]\right) \nonumber \\ \quad= & {} 4\left( \sum _{\omega \in {\mathrm{supp}}\, f}p_{\omega } - \sum _{\omega _A \in \Omega ^{|A|}}p_{\omega _A} \left( \sum _{\omega \in {\mathrm{supp}}\, f}\, p_{\omega | \omega _A}\right) ^2\right) \end{aligned}$$

### Proof

We use the following inequality (found in Topsoe 2001 and used in Heckel et al. 2013) that provides lower and upper bounds on the binary entropy function *h*(*p*)

$$\begin{aligned} 4p(1-p) \le h(p) \le [4p(1-p)]^{1/{\ln 4}}. \end{aligned}$$

If in formula (22) we denote

$$\begin{aligned} q(X_A) = \sum _{\omega \in {\mathrm{supp}}\, f}\, p_{\omega | X _A} \end{aligned}$$

then $\mathrm{LB} = E_A[4q(X_A)(1-q(X_A))]$. For the upper bound, we use the fact that

$$\begin{aligned} E_A[(4q(X_A)(1-q(X_A)))^{1/{\ln 4}}] \le E_A[4q(X_A)(1-q(X_A))]^{1/{\ln 4}}. \end{aligned}$$

Then the double inequality (26) is immediate. Now, LB can be expressed as follows

$$\begin{aligned} \mathrm{LB}= & {} E_A[4q(X_A)(1-q(X_A))]\\= & {} 4E_A\left[ \left( \sum _{\omega \in {\mathrm{supp}}\, f}\, p_{\omega | X _A}\right) \left( 1- \sum _{\omega \in {\mathrm{supp}}\, f}\, p_{\omega | X _A}\right) \right] \\= & {} 4E_A\left[ \left( \sum _{\omega \in {\mathrm{supp}}\, f}\, p_{\omega | X _A}\right) - \left( \sum _{\omega \in {\mathrm{supp}}\, f}\, p_{\omega | X _A}\right) ^2\right] \\= & {} 4\left( \sum _{\omega _A \in \Omega ^{|A|}}p_{\omega _A}\sum _{\omega \in {\mathrm{supp}}\, f}\, p_{\omega | \omega _A} - \sum _{\omega _A \in \Omega ^{|A|}}p_{\omega _A} \left( \sum _{\omega \in {\mathrm{supp}}\, f}\, p_{\omega | \omega _A}\right) ^2\right) \\= & {} 4\left( \sum _{\omega \in {\mathrm{supp}}\, f}p_{\omega } - \sum _{\omega _A \in \Omega ^{|A|}}p_{\omega _A} \left( \sum _{\omega \in {\mathrm{supp}}\, f}\, p_{\omega | \omega _A}\right) ^2\right) \end{aligned}$$

where the first term is obtained by the law of total probability.

$$\square $$

We are interested in identifying possible relationships or inequalities between the mutual information and the influence of a subset of nodes of the network. In Heckel et al. (2013), the authors show that for a given collection of nodes $A \subseteq [n]$, the following holds

28 $$\begin{aligned} I_A(f) \ge \min _{i\in A}\left( \frac{1}{\sigma _i^2}\right) \left[ \mathrm{MI}(f(X);X_A)-\Psi (\mathrm{Var}(f(X)))\right] \end{aligned}$$

where $\Psi (x) = x^{(1/\ln 4)} - x$ takes positive values that are very close to zero, and $\sigma _i^2 = Var(X_i)$, under the assumption of independence of the random variables. Let us explore an alternative inequality using the results of this paper. For this purpose, we consider a special case that allows us to compare directly our results with inequality (28). In this special case, we consider a uniform distribution of inputs, which is actually a case of product distributions or independent inputs.

### Special Case

Consider a network with *n* nodes, such that for each $\omega \in \Omega ^n$, we have $p_{\omega } = \frac{1}{2^n}$, so that we consider a uniform distribution of the inputs. Let $|{\mathrm{supp}}\, f| = K$ and $A \subseteq [n]$. Then

$$\begin{aligned} p_{\omega _A} = P(X_A = \omega _A) = \sum _{\omega _A^c\in \Omega ^{n-|A|}}\frac{1}{2^n} = \frac{1}{2^{|A|}} \qquad \omega _A \in \Omega ^{|A|}. \end{aligned}$$

Using formula (24), we can write the following for the MI,

$$\begin{aligned} \mathrm{MI}(f(X); X_A)= & {} h\left( \sum _{\omega \in {\mathrm{supp}}\, f}\, p_{\omega }\right) - E_A\left[ h\left( \sum _{\omega \in {\mathrm{supp}}\, f}\, p_{\omega | X _A}\right) \right] \\= & {} h\left( \frac{K}{2^n}\right) - \sum _{\omega _A \in \Omega ^{|A|}}p_{\omega _A}h\left( \sum _{\omega \in {\mathrm{supp}}\, f}p_{\omega | \omega _A}\right) \\= & {} h\left( \frac{K}{2^n}\right) - \frac{1}{2^{|A|}}\sum _{\omega _A \in \Omega ^{|A|}}h\left( \sum _{\omega \in {\mathrm{supp}}\, f}p_{\omega | \omega _A}\right) \end{aligned}$$

Note that

$$\begin{aligned} p_{\omega | \omega _A} = P(X = \omega | X_A = \omega _A) = \frac{P(X = \omega , X_A = \omega _A)}{P(X_A = \omega _A)} = \frac{P(X = \omega )}{P(X_A = \omega _A)} = \frac{1}{2^{n-|A|}}. \end{aligned}$$

If we let $K_{\omega _A} =|\hbox {supp }f\cap Pr^{-1}_A(\omega )|$, where $Pr_A$ is the projection of $\omega$ on *A*, then

29 $$\begin{aligned} \mathrm{MI}(f(X); X_A) = h\left( \frac{K}{2^n}\right) - \frac{1}{2^{|A|}}\sum _{\omega _A \in \Omega ^{|A|}}h\left( \frac{K_{\omega _A}}{2^{n-|A|}}\right) . \end{aligned}$$

We immediately notice that $0 \le K_{\omega _A} \le K$ for all $\omega _A \in \Omega ^{|A|}$, and that $\sum _{\omega _A \in \Omega ^{|A|}}K_{\omega _A} = K$, so that we create a partition of ${\mathrm{supp}}\, f$. Therefore, in the sum of (29), some of the terms could be zero, since not all $\omega _A \in \Omega ^{|A|}$ need to be represented in ${\mathrm{supp}}\, f$, leading to $K_{\omega _A} = 0$, which in turn leads to $h(0) = 0$.

Let us focus on $I_A(f) = \sum _{j \in A} I_j(f)$. By formula (18) we obtain

$$\begin{aligned} I_j(f)= & {} \sum _{\omega \in {\mathrm{supp}}\, f\setminus \varphi _j({\mathrm{supp}}\, f)}\left( p_\omega + p_{\varphi _j(\omega )}\right) = \sum _{\omega \in {\mathrm{supp}}\, f\setminus \varphi _j({\mathrm{supp}}\, f)}\frac{1}{2^{n-1}}\\= & {} \frac{|{\mathrm{supp}}\, f\setminus \varphi _j({\mathrm{supp}}\, f)|}{2^{n-1}}. \end{aligned}$$

If $$m_j = |\hbox {supp }f\cap \varphi _j(\hbox {supp }f)|$$ then

$$\begin{aligned} I_j(f) = \frac{K-m_j}{2^{n-1}} \end{aligned}$$

and consequently

30 $$\begin{aligned} I_A(f) = \sum _{j \in A} \frac{K-m_j}{2^{n-1}} = \frac{K|A| - \sum _{j\in A}m_j}{2^{n-1}}. \end{aligned}$$

Notice that $0 \le m_j \le K$ and that $m_j$ is an even number since $\varphi _j$ is its own inverse.

**Fig. 4:**
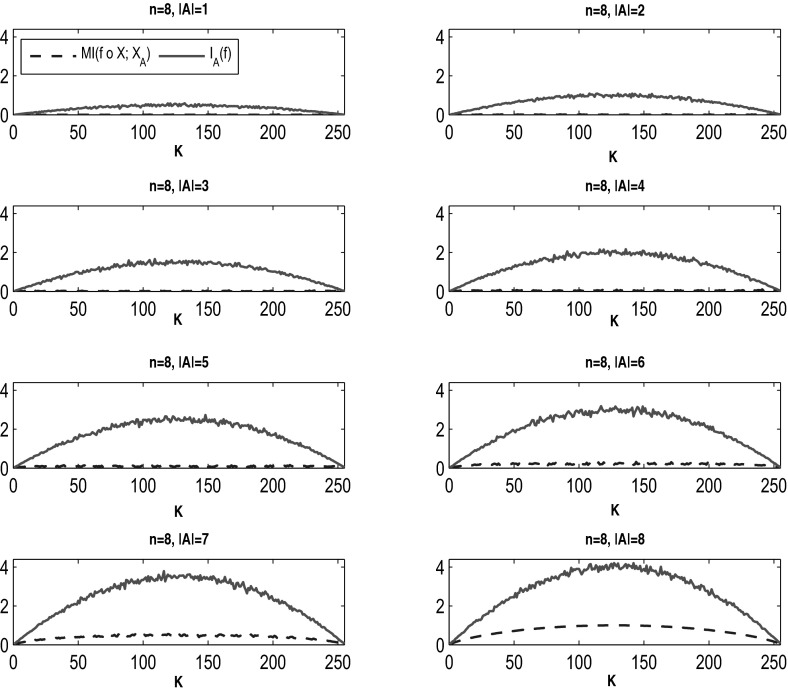
(Color Figure online) Graphs of $I_A(f)$ and $\mathrm{MI}(f(X); X_A)$ computed with formulas (29) and (30) versus *K* for a network with $n = 8$ nodes and $|A| = 1, 2, \ldots , n$ as specified in the subplots. The MI curve is very close to zero for most values of |*A*|, while the influence has much larger values in all cases. Recall also that MI is always a number in [0, 1]. The actual values increase with the increase in |*A*|. Notice also the expected symmetry as *K* crosses from values less than to values greater than $2^n/2$

Observe that if in formula (28) of Heckel et al. (2013) we consider a uniform distribution of random variables, then all $\sigma _i^2 = 1$, and thus the influence bounds above the MI minus a small positive quantity. We would like to check a similar inequality for our case. To this end, we first generate some simulations in which we plot both $\mathrm{MI}(f(X); X_A)$ and $I_A(f)$ versus *K* for various values of |*A*| in Fig. 4, using formulas (29) and (30). Not only is $I_A(f)$ an upper estimate of $MI(f(X); X_A)$, but also its values are significantly larger. So in our case, the inequality becomes

31 $$\begin{aligned} \mathrm{MI}(f(X); X_A) \le I_A(f) \end{aligned}$$

which is stronger than the corresponding inequality of  Heckel et al. (2013). Besides, the quantity $\mathrm{MI}(f(X);X_A)-\Psi (\mathrm{Var}(f(X)))$ used in Heckel et al. (2013) takes on also negative values. For example, one can easily check that this is true for $n = 8, K = 1, |A| = 3, 4, 5$ under the assumptions of the uniform distribution (which leads to independence of random variables). Then the inequality becomes superfluous.

Observe that in this case, if $A = \{ j\}$, and $f_i$ is the Boolean function associated with node *i* of the network, then the inequality (31) becomes $\mathrm{MI}(f_i(X); X_j) \le I_j(f_i)$ which implies $\sum _{i=1}^nMI(f_i(X); X_j) \le \sum _{i=1}^n I_j(f_i)$, or in other words $\mathrm{DP}(j) \le \sigma (f_j)$. However, when looking at the relationship between the DP and strength values in Fig. 2, we observe that this inequality does not hold for all nodes of the *fibroblast* network. That is most likely due to the actual dependencies between the states of the nodes. Thus, dependent inputs may lead to different results. However, we notice in Fig. 2, top right graph, that the magnitudes of the positive differences DP$-\sigma$ are generally smaller than the magnitudes for negative differences. Thus, it may be possible that a version of inequality (28) is still valid. We have not been able to find such an alternative inequality so far. On the other hand, all the examples involving dependent inputs we have looked at, support inequality (31). We provide one such example before returning to the Special Case 3.1.

#### Example 3

Consider the Boolean function *f*(*x*) where $x = (x_1, x_2, x_3)$, represented by the truth table shown below and the corresponding probabilities of states. It is easy to check that this is not a product distribution, so the variables are dependent; for example, $P(X_1=0,X_2=0) = 3/10$ while $P(X_1=0)P(X_2=0) = 1/4$. One can also check that $I_1(f) = 3/5$ and $I_2(f) = I_3(f) = 1/2$. In this case, for any $A \subseteq [3]$ of cardinal at least two, we get automatically that $\mathrm{MI}(f(X); X_A) \le 1 \le I_A(f)$. On the other hand, for $A = \{ 1\}$ we obtain via formula (24), that $\mathrm{MI}(f(X); X_{\{ 1\} }) = h(1/2)-(1/2)h(3/5)-(1/2)h(2/5) = .029 < 0.6 = I_1(f)$. Also, $\mathrm{MI}(f(X); X_{\{ 2\} })= \mathrm{MI}(f(X); X_{\{ 3\} }) = h(1/2)-(1/2)h(7/10)-(1/2)h(3/10) = .1187 < 0.5 = I_2(f) = I_3(f)$. So in all cases, $\mathrm{MI}(f(X); X_A) \le I_A(f)$.

**Table:** 

$(x_1,x_2,x_3)$	$f(x_1,x_2,x_3)$	$P(x_1,x_2,x_3)$
(0, 0, 0)	0	3/20
(0, 0, 1)	1	3/20
(0, 1, 0)	0	1/20
(0, 1, 1)	1	3/20
(1, 0, 0)	1	3/20
(1, 0, 1)	1	1/20
(1, 1, 0)	0	3/20
(1, 1, 1)	0	3/20

Now we return to our Special Case 3.1 and we conjecture that the following inequality is true for all choices of parameters

32 $$\begin{aligned} h\left( \frac{K}{2^n}\right) - \frac{1}{2^{|A|}}\sum _{\omega _A \in \Omega ^{|A|}}h\left( \frac{K_{\omega _A}}{2^{n-|A|}}\right) \le \frac{K|A| - \sum _{j\in A}m_j}{2^{n-1}}. \end{aligned}$$

A general proof of this inequality seems to be very technical and intricate. We present briefly a few particular cases whose proofs can be found in Supplementary Material, Sect. 3 Note that the extreme cases of $K = 0$ and $K = 2^n$ are trivially satisfied since they lead to null quantities on both sides of inequality (32).

**Case 1:** The support is a singleton.

The inequality (32) takes on the particular form

$$\begin{aligned} h\left( \frac{1}{2^n}\right) -\frac{1}{2^{k}}h\left( \frac{1}{2^{n-k}}\right) \le \frac{k}{2^{n-1}} \end{aligned}$$

where $$|A|=k$$.

One obtains the following consequence, which is valid no matter the cardinality of ${\mathrm{supp}}\, f$.

#### Corollary 3

Inequality (32) holds if $|Pr_A(\hbox {supp }\, f)|=1$.

**Case 2:**$${\mathrm{supp}}\, f =\{ \tau ,\eta \} $$, $$\tau \ne \eta $$

The inequality (32) becomes

$$\begin{aligned} h\left( \frac{2}{2^n}\right) -\frac{1}{2^{k}}\sum _{\omega _A\in \Omega ^{k}}h\left( \frac{K_{\omega _A}}{2^{n-k}}\right) \le \frac{2k-\sum _{j\in A}m_j}{2^{n-1}}. \end{aligned}$$

**Case 3:** The support is a subgroup of $\Omega ^n$ and $A=[n]$.

What is meant here is that we identify $\{ 0,1\}$ to $\mathbb {Z}_2$, the additive group of equivalence classes modulo 2, and $\Omega ^n$ to the product group $\mathbb {Z}^n_2$. For any fixed $j\in [n]$, denote by $\delta _j$ the Boolean vector in $\Omega ^n$ whose entries are all null, except entry *j*. Under the previously described identification, one easily sees that, given a Boolean function *f*, the quantities

$$\begin{aligned} m_j=|{\mathrm{supp}}\, f\cap \varphi _j({\mathrm{supp}}\, f)|\qquad j=1,\ldots , n, \end{aligned}$$

can be calculated with the alternative formula

$$\begin{aligned} m_j=|{\mathrm{supp}}\, f\cap (\delta _j+{\mathrm{supp}}\, f)|\qquad j=1,\ldots , n, \end{aligned}$$

where the kind of addition used is addition modulo 2. Finally, recall that the order of a subgroup of $\Omega ^n$ must be a divisor of $2^n$; hence, it will have the form $2^k$ for some nonnegative integer $k\le n$. Keeping all the above in mind, we state and prove the following:

#### Lemma 1

Let *f* be a Boolean function, *S* its support, and $\langle S\rangle$, the subgroup of $\Omega ^n$ generated by *S*. Then, the following inequality holds:

$$\begin{aligned} \sum _{j=1}^nm_j\le k2^k \end{aligned}$$

where $$2^k=|\langle S\rangle |$$.

Now, observe that if $A=[n]$, the inequality we wish to prove has the form

$$\begin{aligned} h\left( \frac{K}{2^n}\right) \le \frac{Kn - \sum _{j=1}^nm_j}{2^{n-1}}. \end{aligned}$$

and if $S={\mathrm{supp}}\, f$ is a subgroup of $\Omega ^n$ of order $2^k$, then the inequality becomes

$$\begin{aligned} h\left( \frac{1}{2^{n-k}}\right) \le \frac{2^kn - \sum _{j=1}^nm_j}{2^{n-1}}. \end{aligned}$$

This one holds since one can write

$$\begin{aligned} h\left( \frac{1}{2^{n-k}}\right) \le \frac{(n-k)}{2^{n-k-1}}=\frac{n2^k-k2^k}{2^{n-1}}\le \frac{2^kn - \sum _{j=1}^nm_j}{2^{n-1}}. \end{aligned}$$

#### Remark 3

If *f* is a Boolean function having support $S, A=[n]$, and $\langle S\rangle \cap \{ \delta _1, \ldots , \delta _n\} =\emptyset$, then (32) holds.

## Final Comments

The main conclusions of this work are that operator theory can offer computationally efficient ways to find or estimate important quantities used in assessing the sensitivity to perturbations of BNs, and to quantify the relevance of nodes using elements of information theory, in particular MI. We conclude that MI is an excellent tool for identifying a subset of relevant nodes in the network that offer the most information gain and whose knowledge reduces the entropy of the whole network significantly. Moreover, the MI provides a lower estimate for the influence of nodes in various scenarios.

It would be of interest to continue this exploration under various scenarios of dependent nodes in the network, as well as to refine further some of the results of this paper. For example, could one strengthen inequality (32) and prove it in general or for different scenarios?

On the other hand, in Klotz et al. (2014) it is shown that MI is maximized for canalizing functions. However, real networks do not consist of a single type of Boolean function. Therefore, it would be of interest to explore a possible hierarchy of various types of functions regarding the information gain they provide. That could offer more information regarding the inequality between the MI and the influence. Besides, most of the functions in real networks, such as cellular networks, need not be strictly canalizing as considered in Klotz et al. (2014) (i.e., one value of one of the inputs forces the output to take on a certain fixed value regardless of the other inputs). In reality, functions may be only partially canalizing, allowing for multiple, but not necessarily all inputs, to be canalizing in a cascading fashion as discussed, for example, in Layne et al. (2012) or Dimitrova et al. (2015). Partially nested canalizing functions have been considered recently as a more realistic alternative to canalizing functions. Another type of function that is common in applications is a threshold function that turns a node ON provided that a sufficient number of inputs are ON. This type of function is typical for neural networks. This opens the door for a variety of directions of research that stem from the work in this paper.

Furthermore, as specified before, it is our intention to explore the Java software Naldi et al. (2009a) to actually perform network reduction preserving attractors and stability and use it to analyze dynamics of various signal transduction networks found in Cell Collective Helikar et al. (2012) and Helikar et al. (2013).

Finally, it would be of great interest to look further into other real networks to identify the possibility of reducing them to the most determinative nodes. For example, it would be interesting to compare the results of the *fibroblast* network to other networks such as the Boolean model of the influenza–host interactions during infection of an epithelial cell Madrahimov et al. (2013), to identify possible similarities and differences that may occur. At the same time, identifying possible classes of biological nodes/sub-networks obtained with the DP sub-network procedure in a variety of other networks could bring further clarifications on the advantages of the DP method. Even more, it is important to assess the degree to which the reduced network provides accurate information on specific tasks typical for the whole network, such as pattern recognition or decision making. At the same time, exploring the impact of considering DP versus strength might provide new insights.

## Electronic supplementary material

Below is the link to the electronic supplementary material.

Supplementary material 1 (pdf 309 KB)
