# Supplementary material for: Logical Reduction of Biological Networks to Their Most Determinative Components
Source: Bull Math Biol. 2016 Jul 14;78:1520–45. doi: 10.1007/s11538-016-0193-x (PMC4993808; doi:10.1007/s11538-016-0193-x)
Supplement: Supplementary file 1 — Supplementary material 1 (pdf 309 KB) [file 11538_2016_193_MOESM1_ESM.pdf]

LOGICAL REDUCTION OF BIOLOGICAL NETWORKS TO THEIR MOST DETERMINATIVE COMPONENTS: SUPPLEMENTARY MATERIAL

MIHAELA T. MATACHE* AND VALENTIN MATACHE

Department of Mathematics
University of Nebraska at Omaha
Omaha, NE 68182-0243, USA
dmatache@unomaha.edu, vmatache@unomaha.edu
* corresponding author

This supplementary material contains further information on some of the topics contained in the paper, as well as some proofs. All labels and formula numbers refer to the corresponding ones in the main manuscript.

1. Computational aspects

This section is related to the last part of Section 2 of the paper. We recall all relevant information from the paper.

Now let us turn to the computational aspects of formulas (14) and (18). Recall that, if k_i is the connectivity of node i, we need $i \times k_i \times 2^{2k_i}$ "for loops" for computing formula (14) in Matlab, while for formula (18) there are only $i \times k_i$ loops which is clearly a much smaller number (no parallel computing is used). To assess graphically the magnitude of the differences, we measure the CPU time needed to compute influences for the nodes of a given network by the two formulas. We do this for identical sub-networks of increasing sizes, to determine the impact of the network size.

Recall that the network under consideration is the signal transduction network of a generic *fibroblast* cell which consists of several main signaling pathways, including the receptor tyrosine kinase, the G-protein coupled receptor, and the integrin signaling pathway.

For our purposes, we generate sub-networks of increasing sizes by simply adding nodes in alphabetic order. Thus we measure the CPU time and plot it against the network size in the top panel of Figure 1. We also compute the corresponding average and maximum connectivities for each network scenario, and plot them against the networks size in the bottom panel of Figure 1. We note that with formula (14), labeled as "Formula 1" in the figure, it is prohibitive to consider connectivities greater than 10, since the CPU time increases exponentially, as seen in both panels around network size 16, while the situation is totally different for

formula (18), labeled as "Formula 2", which yields significantly lower CPU times. The CPU time "explodes" once the maximum connectivity reaches 10 for formula (14).

Using formula (18) we could actually keep increasing the sub-network sizes until we reach the full network without worrying about connectivity. We plot the results in Figure 2 where we note the linear increase of the CPU time with network size. Thus we conclude that formula (18) is significantly more computationally efficient than formula (14).

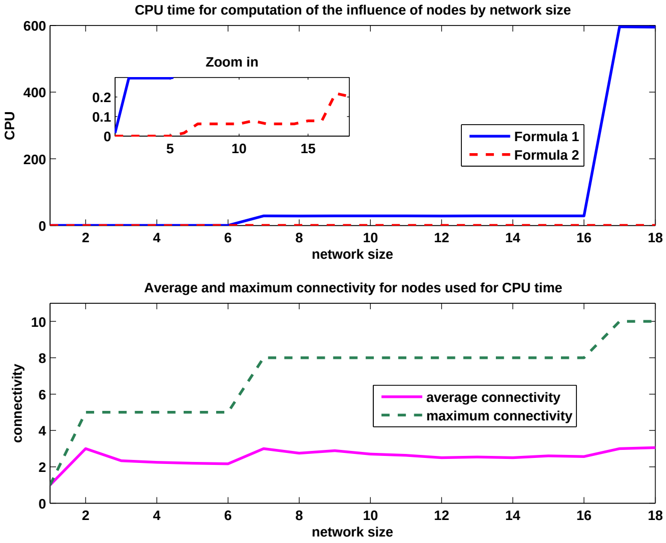

Figure 1. Top panel: CPU time (in seconds) computed with formula (14), labeled as "Formula 1", and with formula (18), labeled as "Formula 2", plotted against increasing networks size. Bottom panel: the corresponding average and maximum connectivity in the sub-networks of the given sizes. Due to computational limitations, the maximum network size is 18 and the maximum connectivity 10.

2. Statistical analysis

This section is related to Section 3 of the paper. We recall the information from the manuscript with related figures.

We have conducted a statistical analysis related to DP and σ values for the *fibroblast* network. In summary, there is enough statistical evidence that the average DP-σ is negative with a p-value of basically zero. The paired test gives an upper bound of -0.14208 for a 95% confidence interval for the difference DP$-\sigma$. On the other hand a linear regression analysis indicates a fairly strong linear relationship between the two variables with a 75.1% coefficient of determination (COD), Figure 4, and a higher COD of 82.4% for the linear relationship between the average σ and the number of outlinks corresponding to the nodes, Figure 5. The average values are computed over all nodes with a given number of outlinks. This relationship is weaker for average DP versus number of outlinks with a COD of 60.3%, Figure 6. We also note that the outliers occur mostly for nodes with a larger number of outlinks. In other words, fewer outlinks generate a stronger correlation between the DP or σ and the number of outlinks.

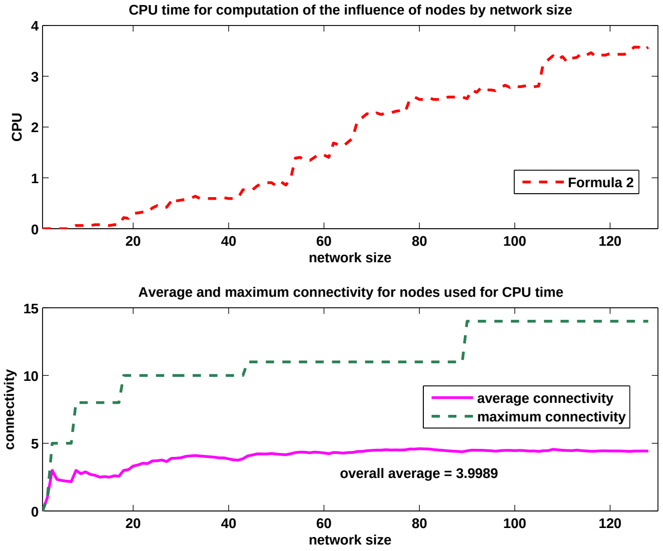

FIGURE 2. Top panel: CPU time (in seconds) computed with formula (18), labeled as "Formula 2", plotted against increasing networks size. Bottom panel: the corresponding average and maximum connectivity in the sub-networks of the given sizes. Due to the computational efficiency of the formula, the network size is increased to the maximum possible of 130, and the maximum connectivity is 14, as indicated in the description of the *fibroblast* network.

For example, there is one particular node in the network, namely *EGFR*, that generates the maximum DP and is the only node with 13 outlinks. If we eliminate this node from the correlation analysis, the COD for average DP versus outlinks increases from 60.3% to 81.3%. Notably, mutations of the *EGFR*, epidermal growth factor receptor, are known to be related to lung cancer, interfering with the signaling pathways within the cell triggered to promote cell growth and division (proliferation) and cell survival. The second node in the order of DP is *ASK1*, apoptosis signal-regulating kinase 1, and plays important roles in many stress-related diseases, including cancer, diabetes, cardiovascular and neurodegenerative diseases. The third node is *Src*, proto-oncogene tyrosine-protein kinase, is involved in the control of many functions, including cell adhesion, growth, movement and differentiation. The fourth node is *PIP3_345*, Phosphatidylinositol (3,4,5)-trisphosphate, that functions to activate downstream signaling components, while the fifth node is *PKC*, protein kinase C, involved in receptor desensitization, in modulating membrane structure events, in regulating transcription, in mediating immune responses, in regulating cell growth, and in learning and memory. The DP procedure managed to capture the importance of these nodes in relationship to the rest of the network. Four of the top five DP nodes, are also among the five strongest nodes which are: *Src*, *PIP3_345*, *PKC*, *PIP2_45*, and *EGFR*. Thus, the strength also captures biologically important nodes.

Moreover, higher DP and strength values are correlated with a larger number of outlinks as seen from the figures, which means that this procedure can identify hubs in the network. It is also apparent from the figures that the COD increases when considering smaller DP and σ values.

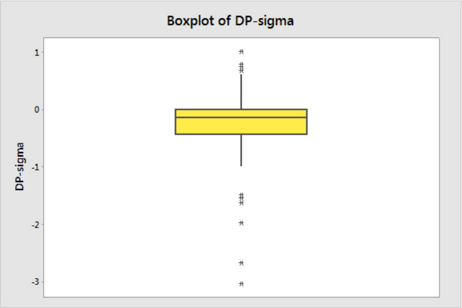

FIGURE 3. Boxplot for the DP-σ. The statistical test on paired means indicates that there is enough evidence that the mean of DP -σ is negative with a p-value of basically zero.

3. Special cases for the inequality on MI and influence

This section is related to the last part of Section 3 of the paper. We recall all relevant information from the paper.

We conjecture that the following inequality is true for all choices of parameters

$$h\left(\frac{K}{2^n}\right) - \frac{1}{2^{|A|}} \sum_{\omega_A \in \Omega^{|A|}} h\left(\frac{K_{\omega_A}}{2^{n-|A|}}\right) \leq \frac{K|A| - \sum_{j\in A} m_j}{2^{n-1}}. \tag{37}$$

A general proof of this inequality seems to be very technical and intricate as can be seen in the next few cases. Note that the extreme cases of $K = 0$ and $K = 2^n$ are trivially satisfied since they lead to null quantities on both sides of inequality (37).

Case 1: The support is a singleton.

The inequality (37) takes on the particular form

$$h\left(\frac{1}{2^n}\right) - \frac{1}{2^k} h\left(\frac{1}{2^{n-k}}\right) \leq \frac{k}{2^{n-1}} \tag{38}$$

where $|A| = k$.

It is our aim to prove that

$$0 \leq \frac{h(x)}{2^k} + \frac{kx}{2^{k-1}} - h\left(\frac{x}{2^k}\right) \qquad 0 \leq x \leq 1. \tag{39}$$

As one can see, once (39) is proved, substitution of x by $1/2^{n-k}$ in (39) leads to (38). Using calculus methods, denote

$$F(x) = \frac{h(x)}{2^k} + \frac{kx}{2^{k-1}} - h\left(\frac{x}{2^k}\right) \qquad 0 \leq x \leq 1.$$

The equation $F'(x) = 0$ is equivalent to

$$\frac{k}{2^{k-1}} = \frac{\ln\left(\frac{2^k - x}{1-x}\right)}{2^k \ln 2}$$

which has the solution

$$x_k = \frac{4^k - 2^k}{4^k - 1}$$

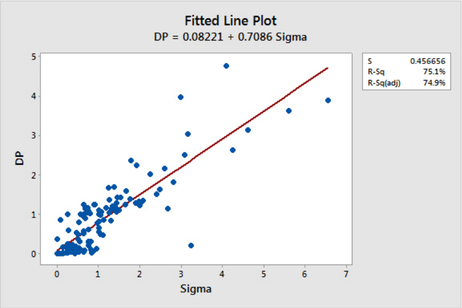

FIGURE 4. Linear regression fit for DP versus σ. Note that most outliers occur for larger values of DP and σ.

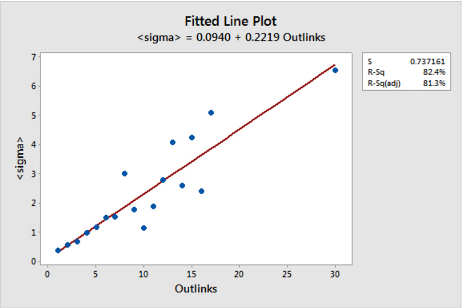

FIGURE 5. Linear regression fit for average σ versus number of outlinks (averages over all available nodes with each given number of outlinks).

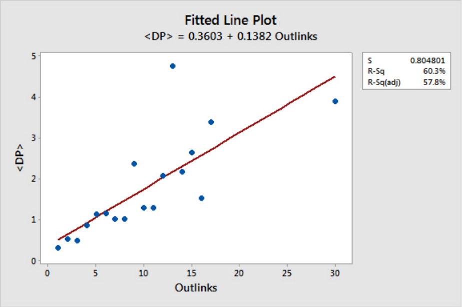

FIGURE 6. Linear regression fit for average DP versus number of outlinks (averages over all available nodes with each given number of outlinks). Upon elimination of the outlier corresponding to 13 outlinks, the coefficient of determination becomes 81.3%.

and it is easy to see that $F' > 0$ on interval $(0, x_k)$, and $F' < 0$ on interval $(x_k, 1)$. Thus F increases from $F(0) = 0$ to $F(x_k)$, then decreases from $F(x_k)$ to $F(1)$, and so, if we prove $F(1) \geq 0$, one gets $F(x) \geq 0$, $0 \leq x \leq 1$, that is (39) holds.

To finish, note that the inequality $F(1) \geq 0$ is equivalent to

$$h\left(\frac{1}{2^k}\right) \leq \frac{k}{2^{k-1}} \qquad (40)$$

which is the particular form of (38) when $n = k$.

It is straightforward to show that the inequality (40) is equivalent to

$$k - \log_2(2^k - 1) \leq \frac{k}{2^k - 1}. \tag{41}$$

Denoting $x = 2^k - 1$, note that $k - \log_2(2^k - 1) = \log_2(x+1) - \log_2 x = \frac{1}{c \ln 2}$ for some $x < c < x + 1$, by the mean value theorem. Thus

$$k - \log_2(2^k - 1) \leq \frac{1}{x \ln 2} = \frac{1}{\ln 2(2^k - 1)} \leq \frac{k}{2^k - 1}, \qquad k \geq \frac{1}{\ln 2}$$

so the inequality (41) holds for $k = 2, 3, \ldots$. It also holds for $k = 1$, since in that case, it simplifies to $1 \leq 1$.

One obtains the following consequence of the above estimates, which is valid no matter the cardinality of supp$f$:

Corollary 1. *Inequality* (37) *holds if* $|Pr_A(\text{supp} f)| = 1$.

Indeed, in that case K_{ω_A} is null for all but one value of ω_A. If $Pr_A(\text{supp} f) = \omega_A$, then $K_{\omega_A} = K$. Thus, the inequality becomes

$$h\left(\frac{K}{2^n}\right) - \frac{1}{2^k} h\left(\frac{K}{2^{n-k}}\right) \leq \frac{Kk}{2^{n-1}}. \tag{42}$$

Indeed $m_j = 0$ for all $j \in A$, in our case. Note that $K \leq 2^{n-k}$. Letting $x = K/2^{n-k}$ in (39) one obtains (42).

Case 2: supp$f = \{\tau, \eta\}$, $\tau \neq \eta$

The inequality (37) becomes

$$h\left(\frac{2}{2^n}\right) - \frac{1}{2^k} \sum_{\omega_A \in \Omega^k} h\left(\frac{K_{\omega_A}}{2^{n-k}}\right) \leq \frac{2k - \sum_{j \in A} m_j}{2^{n-1}}. \tag{43}$$

Subcase (a) The vectors τ and η are different in 2 slots ore more. In that case $m_j = 0$ for all $j \in A$. Thus, we want to show

$$h\left(\frac{1}{2^{n-1}}\right) - \frac{1}{2^k} \sum_{\omega_A \in \Omega^k} h\left(\frac{K_{\omega_A}}{2^{n-k}}\right) \leq \frac{k}{2^{n-2}}. \tag{44}$$

If $Pr_A(\eta) \neq Pr_A(\tau)$, then $K_{\omega_A} = 1$ if $\omega = \tau$ or $\omega = \eta$. Thus, one needs to show

$$h\left(\frac{1}{2^{n-1}}\right) - \frac{1}{2^{k-1}} h\left(\frac{1}{2^{n-k}}\right) \leq \frac{k}{2^{n-2}}. \tag{45}$$

If one substitutes n by $n-1$ and k by $k-1$ in (38), one gets

$$h\left(\frac{1}{2^{n-1}}\right) - \frac{1}{2^{k-1}} h\left(\frac{1}{2^{n-k}}\right) \leq \frac{k-1}{2^{n-2}} < \frac{k}{2^{n-2}}, \tag{46}$$

hence (45) holds.

If $Pr_A(\eta) = Pr_A(\tau)$, then the inequality holds since $|Pr_A(\text{supp} f)| = 1$.

Subcase (b) The vectors τ and η are different in just 1 slot, say slot j. In that case one needs to prove

$$h\left(\frac{1}{2^{n-1}}\right) - \frac{1}{2^k} \sum_{\omega_A \in \Omega^k} h\left(\frac{K_{\omega_A}}{2^{n-k}}\right) \leq \frac{k-1}{2^{n-2}} \tag{47}$$

if $Pr_A(\eta) \neq Pr_A(\tau)$. Then $K_{\omega_A} = 1$ if $\omega = \tau$ or $\omega = \eta$ leading to

$$h\left(\frac{1}{2^{n-1}}\right) - \frac{1}{2^{k-1}} h\left(\frac{1}{2^{n-k}}\right) \leq \frac{k-1}{2^{n-2}}, \tag{48}$$

which holds by (46).

If $Pr_A(\eta) = Pr_A(\tau)$, the inequality holds since $|Pr_A(\text{supp}f)| = 1$.

Case 3: The support is a subgroup of Ω^n and $A = [n]$.

What is meant here is that we identify $\{0,1\}$ to $\mathbb{Z}_2$, the additive group of equivalence classes modulo 2, and Ω^n to the product group $\mathbb{Z}_2^n$. For any fixed $j \in [n]$, denote by δ_j the Boolean vector in Ω^n whose entries are all null, except entry j. Under the previously described identification, one easily sees that, given a Boolean function f, the quantities

$$m_j = |\text{supp}f \cap \varphi_j(\text{supp}f)| \qquad j = 1, \ldots, n,$$

can be calculated with the alternative formula

$$m_j = |\text{supp}f \cap (\delta_j + \text{supp}f)| \qquad j = 1, \ldots, n,$$

where the kind of addition used is addition modulo 2. Finally, recall that the order of a subgroup of Ω^n must be a divisor of 2^n, hence it will have the form 2^k for some nonnegative integer $k \leq n$. Keeping all the above in mind, we state and prove the following:

Lemma 1. *Let f be a Boolean function, S its support, and $\langle S \rangle$, the subgroup of Ω^n generated by S. Then, the following inequality holds:*

$$\sum_{j=1}^{n} m_j \leq k2^k \tag{49}$$

where $2^k = |\langle S \rangle|$.

Proof. For any fixed $j \in [n]$, one has

$$m_j = |\text{supp}f \cap (\delta_j + \text{supp}f)| \leq |\langle S \rangle \cap (\delta_j + \langle S \rangle)|.$$

The sets $\langle S \rangle$ and $(\delta_j + \langle S \rangle)$ are equivalence classes of Ω^n modulo $\langle S \rangle$, and hence either coincide or are disjoint. The aforementioned classes are disjoint if and only if $\delta_j \notin \langle S \rangle$, in which case $m_j = 0$. Thus, if one denotes by E the subset of $[n]$ consisting of those numbers j, that satisfy $\delta_j \in \langle S \rangle$, then, one has that

$$\sum_{j=1}^{n} m_j = \sum_{j \in E} m_j \leq |E||\langle S \rangle| = |E|2^k.$$

On the otther hand, the set $M = \{\delta_j : j \in E\}$ generates a subgroup of $\langle S \rangle$ of order $2^{|E|}$, hence $|E| \leq k$ and so, (49) holds. □

Now, observe that if $A = [n]$, the inequality we wish to prove has the form

$$h\left(\frac{K}{2^n}\right) \leq \frac{Kn - \sum_{j=1}^{n} m_j}{2^{n-1}}. \tag{50}$$

and if S =supp$f$ is a subgroup of Ω^n of order 2^k, then inequality (50) becomes

$$h\left(\frac{1}{2^{n-k}}\right) \leq \frac{2^k n - \sum_{j=1}^{n} m_j}{2^{n-1}}. \tag{51}$$

We claim that (51) holds. Indeed, by (40) and Lemma 1, one can write

$$h\left(\frac{1}{2^{n-k}}\right) \leq \frac{(n-k)}{2^{n-k-1}} = \frac{n2^k - k2^k}{2^{n-1}} \leq \frac{2^k n - \sum_{j=1}^{n} m_j}{2^{n-1}}.$$

Remark 1. *If f is a Boolean function having support S, $A = [n]$, and $\langle S \rangle \cap \{\delta_1, \ldots, \delta_n\} = \emptyset$, then* (37) *holds.*

Indeed, by the proof of Lemma 1, in this case, one has that $\sum_{j=1}^{n} m_j = 0$ and so, what one has to prove is that

$$h\left(\frac{K}{2^n}\right) \leq \frac{nK}{2^{n-1}},$$

a fact that follows if one shows that

$$F(x) = \frac{nx}{2^{n-1}} - h\left(\frac{x}{2^n}\right) \geq 0 \qquad 1 \leq x \leq 2^n.$$

The above inequality holds, since one checks easily that $F'(x) \geq 0$ if $1 \leq x < 2^n$ and

$$F(1) = \frac{n}{2^{n-1}} - h\left(\frac{1}{2^n}\right) \geq 0,$$

by (40).
